# Trends in National R&D Projects on Biomimetics in South Korea

**DOI:** 10.3390/biomimetics10050275

**Published:** 2025-04-29

**Authors:** Hyein Na, Eunhee Kim

**Affiliations:** 1 Department of Science and Technology Management Policy, University of Science and Technology, 217 Gajeong-ro, Yuseong-gu, Daejeon 34113, Republic of Korea; nanahi@ust.ac.kr; 2 College of Business Administration, Chonnam National University, 77 Yongbong-ro, Buk-gu, Gwangju 61186, Republic of Korea

**Keywords:** biomimetics, R&D projects, network analysis, topic modeling, innovation

## Abstract

Imitating nature’s mechanisms has enormous potential to improve our lives and tools. Biomimetics emulates nature’s proven patterns and strategies to develop novel solutions widely applied in various fields. This study aims to propose an overall perspective and research direction for innovation using biomimetics. Using text network analysis and topic modeling, we analyzed the evolution of 5202 Korean R&D projects in biomimetics. The results indicate significant interdisciplinary collaborations between bioengineering, drug development, polymer chemistry, and robotics. Moreover, biomimetic national R&D has primarily focused on fundamental research and its trends reveal interconnection with topic clusters around intelligent robotics, biomedical engineering, and materials science. This study provides guidelines for governments and R&D organizations to establish biomimetic R&D plans and select convergence topics for innovation.

## 1. Introduction

Biomimetics is gaining worldwide attention as an important mechanism to address societal issues such as climate change, environmental pollution, and the transition to carbon-neutral energy systems. It is an innovative approach strategically derived from nature’s ecosystems, which have evolved to adapt to their environment [1,2]. This approach facilitates sustainable development through the application of biodiversity and environmentally benign properties into products, processes, and systems across multiple industries covering healthcare [3], robotics [4], architecture [5], and manufacturing [6]. Biomimetics has significant potential to enhance the circular economy model and affect the advancement of green technologies, all while reducing environmental impact and preserving energy efficiency [7,8].

Biomimetics provides opportunities for sustainable development through efficient resource management, reduced environmental impact, and value creation in emerging industries. The Fermanian Business & Economic Institute (FBEI) reported that the Da Vinci Index, which tracks biomimetics-related papers, patents, and grants, increased twelvefold between 2000 and 2019. The global biomimetics market size is expected to reach approximately $1.6 trillion by 2030, creating about 2.4 million jobs across a variety of sectors, including transportation, architecture, electronics, and data platforms [9,10].

Countries such as the United States, Germany, and France view biomimetics as a promising field that can innovate new industries while addressing environmental issues, including climate change and carbon neutrality [11,12,13]. These countries actively promote the advancement of biomimetic technologies through research investment and policy programs [11]. Their support ranges from conducting basic research to investigating the structural characteristics of living organisms and from facilitating the commercialization of products to enabling their entry into new markets. For instance, the United States focuses on defense and robotics, strategically allocating resources to priority biomimetic applications [13,14]. In Germany, to promote technological advancement and expand research areas, collaborative biomimetic research networks (BIOKON and Kompetenznetz Biomimetik) have been established with firms and research institutes. France promotes technology diffusion and cross-section collaboration in biomimetics through the flagship event ‘Biomim’EXPO [12,15].

Previous studies on biomimetic research trends [16,17,18,19,20] have identified patterns of growth, leading countries, and key contributors by quantitatively analyzing publications or patents. These studies have primarily focused on individual technologies or specific research achievements, conducting bibliometric analysis, literature reviews, and case studies. To deepen our understanding of biomimetic research trends, it is necessary to analyze cross-disciplinary convergence, temporal shifts in topics, thematic clusters, and pathways from science/technology to commercialization. National funding in biomimetic fields also has difficulty in establishing well-balanced strategies due to a lack of scientific data for trends in biomimetic research over time. Accordingly, there is a need for more comprehensive and integrated investigations of biomimetics in terms of a macroscopic context. Text-mining analytical tools for a large set of biomimetic research data can provide the visualization and understanding of the biomimetic research networks. In other words, combining a wide set of text-mining techniques—spanning co-interdisciplinary analysis, keyword analysis, and topic modeling—can contribute to mapping out the emerging intellectual structure [21] of the innovation pertaining to biomimetics. In addition, the outcomes by systematically illustrating research trends can provide a body of knowledge and comprehensive insights into the types and characteristics of research projects that drive the transition of biomimetic technologies from the laboratory to industrialization.

The aim of this study is to investigate biomimetic R&D trends to provide future research directions and R&D policy implications. Hence, we address a hypothetical question to identify key driving factors for the evolution of biomimetic research based on research keywords and interdisciplinary areas. For this purpose, we employed text-mining techniques to analyze the research structure and trends of biomimetic R&D projects conducted in South Korea from 2009 to 2023. Further, we explored how the amount of R&D funding has changed over time and what the primary interdisciplinary research areas are within their network structure patterns. The present study may provide policymakers and enterprises with insights into the evolution and potential areas of biomimetic R&D.

## 2. Literature Review

### 2.1. Biomimetics

#### 2.1.1. Definition

The term “biomimetics” was initially coined by neurophysiologist Otto H. Schmitt in 1957. It originates from the Greek words “bios”, meaning life, and “mimesis”, meaning imitation [2]. Biomimetics seeks to understand natural mechanisms and discover sustainable solutions by mimicking various biological structures, behaviors, and systems. In addition, it involves learning from and emulating natural ecosystems that have been tested by the environment and refined through evolution, as well as the design and fabrication of multifunctional materials and devices of commercial interest [22]. Applying ecological systems and nature’s excellent properties to products and services can create new technological innovations and sustainable solutions [23,24,25].

In her book, published in 1997, ecologist Janine Benyus introduces the word “biomimicry” as “innovation inspired by nature”, emphasizing that biomimetics paves the way to new technological development by emulating optimal ideas and designs from nature [1]. Additionally, biomimetics is described as a field that will help solve societal problems, such as reducing pollution and increasing energy efficiency. As a biological strategy, biomimetic technology has been utilized, for example, in self-cleaning surfaces inspired by lotus leaves, in efficient propulsion systems inspired by jellyfish, and in wind turbine blades that mimic whale fins [26]. Biomimetics is a highly interdisciplinary approach that integrates biology and technology by transforming nature’s principles into a technological solution [27]. It refers to leveraging ecological systems as models for innovative technologies, drawing on nature to address the social, environmental, and economic challenges of sustainable development. Similar terms such as biomimetics, biomimicry, bionics, bioinspiration, and nature-based solutions have been introduced to describe the process through which nature principles inspire technical solutions [28,29]. This study exclusively uses the term “biomimetics” throughout the present article, while acknowledging the conceptual similarity among these terms.

#### 2.1.2. Approaches for Sustainable Innovation

Biomimetics, an interdisciplinary approach inspired by living beings in nature, offers sustainable, efficient, and innovative solutions to a wide range of human problems by leveraging principles observed in natural systems. This approach goes beyond simple improvement of existing methods and proposes new strategies with the transformative potential to reshape industries, advance technologies, and refine management practices. Moreover, the flow of ideas and functional principles from nature to technology can foster the advancement of sustainable innovation via biomimetic solutions [30,31].

Generally, biomimetics processes follow two approaches: “biology push” and “technology pull”. The biology push approach involves discovering effective structures, functions, systems, and other features from natural ecosystems and linking them to technological development. This approach is a “biomimetic development process where the knowledge gained from basic research in the field of biology is used as the starting point and applied to the development of new technical products” [27]. This approach requires that specific functions or behaviors of organisms or natural ecosystems first be identified through extensive biological research and then transferred to the design to influence it. Furthermore, it has been referred to as the “Solution-based approach”, “Biology influencing design”, and “Bottom-up method” [32,33,34]. The development of Velcro tape and waterproof paints and coatings constitute notable examples of the biology push approach. Velcro tape was inspired by the hook shape of the cocklebur [35], while waterproof paints and coatings were derived from the study of leaf surface tension [36,37].

The technology pull approach presents nature-inspired sustainable solutions to address challenges or functionalities of products that are not addressed by existing technologies. This approach is defined as a “biomimetic development process wherein an existing functional technical product is provided with new or improved functions through the transfer and application of biological principles” [27]. This approach is also known as the “problem-based”, “design looking to biology”, and “top-down approach” [33,34,38,39], where designers initially study how organisms solve problems in their natural environments or ecosystems to find analogous solutions and then apply them to a technology or product. An example of the technology pull approach is the development of a new model to reduce noise on Shinkansen high-speed trains in Japan, inspired by studying the head shape and beak angle of kingfishers [40].

Researchers are paying attention to finding ways to translate biomimetics into marketable prototypes, viable products, and new processes. This includes its role in driving technological, scientific, and business innovation [41], as well as its broader impact on the economy [11]. Biomimetics has found new applications in areas such as advanced materials and surface engineering [42,43,44], fluid dynamics and robotics [45,46], and even architecture/building services [47,48,49]. Currently, biomimetics provides not only this growing feasibility in the aspect of a methodology but also a useful tool through which companies develop innovative strategies affecting technologies, supply chains, and organizational processes [50,51]. For instance, TRIZ (Theory of Inventive Problem Solving) is the most commonly used biomimetic tool that helps designers systematically solve problems by mimicking natural systems [52]. Some tools, including AskNature [53], BioTRIZ [54], E2B-Thesaurus [55], BIDARA [56], and BiOMIg Search (integrated into Asteria) [57], support various stages of the biomimetic design process—from ideation to biological abstraction and technical transfer—offering accessibility, interdisciplinary compatibility, and AI-enhanced capabilities [58].

### 2.2. Text Mining

Text mining refers to the process of discovering new, unknown patterns or knowledge from a large volume of unstructured text data using natural language processing technology, information retrieval, and extraction [59,60,61]. Text mining extracts potential subjects and major agendas by identifying the relationships among the keywords mentioned in documents and clustering similar words [62,63]. Text classification and clustering, keyword analysis, network analysis, thematic analysis, and sentiment analysis are examples of specific analytical methods that can be used to discern important discussions and meaningful information in the text. Recently, policymakers have adopted text mining, which integrates network analysis and text analysis, as a new decision-making tool using diverse datasets from academic papers, patents, news articles, and social media platforms [64].

#### 2.2.1. Text Network Analysis

Text network analysis (TNA), a variant of social network analysis (SNA), is one of the techniques used in text analysis. SNA is a scientific and quantitative method for analyzing the relational properties of social phenomena occurring among individuals, organizations, and groups [65]. The structure of a social network consists of nodes and links based on graph theory. This method focuses on analyzing the connections between nodes and the characteristics of the links [66]. To understand the underlying meaning of the text, TNA explores the relationships between interconnected words through the structural arrangement and co-occurrence of words within an entire text [67]. The influence of words in the network is assumed to be substantial when two different words appear frequently in the same text and are located close to each other [68].

Centrality is an indicator that quantifies the influence of nodes in the network. Metrics such as degree centrality, closeness centrality, betweenness centrality, and eigenvector centrality are usefully utilized to ascertain the role and relative importance of nodes [69,70]. In text networks, these metrics facilitate the identification of core terms and their interconnections, offering a more profound comprehension of the text’s semantic and structural framework. TNA is highly effective in visualizing connections among influential terms within extensive document collections, thereby revealing intricate patterns in their relationships [67,71,72]. TNA application in research analysis facilitates the explanation of research trend evolution, forecasting of future issues, and exploration of textual knowledge structures. This method further provides valuable capabilities for quantifying conceptual relationships and identifying research patterns through knowledge structure visualization analysis [73,74]. Given these advantages, this study employed TNA to examine the structural configurations of two separate networks in biomimetic R&D, one constructed from research keywords and the other from interdisciplinary research fields.

#### 2.2.2. Topic Modeling

Topic modeling has gained attention as a powerful text-mining technique and one of the most popular probabilistic clustering algorithms [75]. This method is based on the concept that word frequency patterns characterize specific topics within a given text, as discussed by various researchers [76,77]. Topic modeling can provide a new perspective for predicting technological changes and a complementary method for identifying potential topics concealed in hundreds of documents [78,79]. Recently, science and technology studies have applied topic modeling to group words with the purpose of outlining the underlying topics beyond those words and predicting breakthrough innovations. By analyzing technology and industry trends, such as those of artificial intelligence (AI) [80], blockchain [81], energy [82], healthcare [83], and solar cells [84], it has been used to discover technical challenges and guide policy planning.

Table 1, adapted from Refs. [85,86], compares the different types of topic models available in studies across strengths and limitations. Latent Dirichlet Allocation (LDA) is a representative unsupervised learning topic model that functions as a probabilistic generative model to analyze text document corpora. It assumes that documents consist of a mixture of latent topics, each topic represented as a probability distribution over words [87,88,89]. Topic structures in large volumes of textual data can be derived quickly and efficiently using the LDA model [78].

LDA was selected for this study based on its strong performance across eight comparative dimensions. One notable feature of LDA is its effective adaptable capability to new document collections not present in training datasets [85]. While models such as TF-IDF (Term Frequency-Inverse Document Frequency) depend primarily on statistical word patterns and document distinctiveness without capturing semantic relationships [90,91], LDA offers the extraction of underlying conceptual frameworks from text collections. When contrasted with alternative models like NMF (Non-negative Matrix Factorization) [92,93,94], LDA’s statistical modeling approach shows particular adaptability to textual diversity [95]. While NMF provides thematic clustering of comparable quality, LDA’s distinct advantages in interpretability and its probabilistic structure offer superior clarity when visualizing content themes within analyzed materials. LDA is founded on a fundamentally different mathematical framework compared to models such as TF-IDF, NMF, and BERTopic [96]. Comparative studies have presented the possibility that LDA can produce topic modeling results comparable to those generated by these models [97]. Accordingly, LDA was applied due to its effectiveness in analyzing extensive science and technology data, enabling intuitive comprehension of content and facilitating the tracking of topic evolution over time.

## 3. Methodology

**Figure 1:**
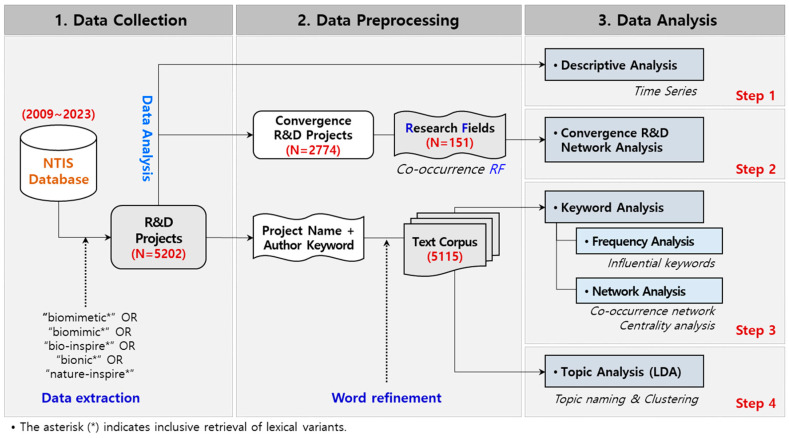
Research model.

The overall process of this study is depicted in Figure 1. It comprises several steps to analyze biomimetic research trends and key topics. The open-source software, R, which provides many packages for text analysis, was used, along with Gephi (version 0.10.1) and VOSviewer (version 1.6.20) for network analysis and visualization.

### 3.1. Data and Preprocessing

Data on biomimetics-related R&D projects were obtained from the National Science and Technology Information Service (NTIS), an integrated national R&D database managed by the Korea Institute of Science and Technology Information (KISTI) in South Korea [98]. NTIS provides integrated R&D information on programs, financial, performance, and human resource data in South Korea. Data were retrieved from the NTIS database utilizing search terms “biomimetic*”, “biomimic*”, “bio-inspir*”, “bionic*”, and “nature-inspir*”, as standardized by the International Standardization Organization (ISO). The dataset comprised 5202 R&D projects of biomimetics conducted between 2009 and 2023. The extracted data were filtered, consisting of ten essential attributes: project title, year, National Science and Technology Standard Classification (NSTSC), technology classification, application field, R&D stage, executing institution, government funding, research content, and research keywords.

The preprocessing involves two phases. First, textual data underwent refinement processes: (1) merging “project titles” with “research keywords”; (2) utilizing computational text analysis tools within R—”NLP4kec” and “tm”—to extract nouns and eliminate superfluous elements (e.g., digits, punctuation, special characters, prepositions, and stopwords); (3) standardizing synonyms and abbreviations possessing identical semantic content despite different lexical forms; and (4) excluding commonly recurring words (e.g., research, analysis, study, development, etc.) typical of scientific documentation. The refined lexical elements were used as primary analytical inputs for keyword network and thematic analysis, given their function as salient indicators of the research focus. Second, interdisciplinary convergence projects were identified based on the research field classifications defined within the NSTSC. Through this preprocessing procedure, we extracted 2774 R&D projects representing multiple research fields and we derived characteristic co-occurrence pairs of convergent research fields from them.

### 3.2. Research Process

A four-step data analysis process was systematically applied to understand trends in biomimetic research. In the first step, we conducted a descriptive statistical analysis of the number of R&D projects and research funds over time to analyze trends in biomimetics. Next, an interdisciplinary research network was analyzed to identify clusters and convergence relationships among research fields. We described the convergence areas of biomimetic research that play an important role in advancing interdisciplinary collaboration. The network represented research fields as nodes, and the strength (weight) of each link depicts the frequency of their co-occurrences. In addition, we evaluated the network’s structural connectivity using four centrality measures: degree centrality, closeness centrality, betweenness centrality, and eigenvector centrality.

Keyword analysis compared the time evolution of common and emerging keywords divided into three periods: 2009–2013, 2014–2018, and 2019–2023. In addition, we visualized a keyword network by co-occurring words (nodes), connectivity among words (edges), and their frequency (weights). A network centrality analysis was conducted to evaluate the most influential keywords and their relative significance. More specifically, we presented our findings from each biomimetics keyword cluster to gain insight into recent research trends.

Finally, latent research themes were identified using LDA topic modeling, combined with hierarchical clustering represented by dendrograms. To determine the optimal number of topics (k), four statistical metrics—Griffiths2004, Deveaud2014, Arun2010, and CaoJuan2009—were calculated for a range of topic counts (k = 2 to 20) using the “ldatuning” R package [99]. While each of the four evaluation metrics indicated a shift in trend, this value did not necessarily correspond to global extrema across all curves; it represented a region in which the trade-off between model parsimony and topic distinctiveness becomes more balanced. Therefore, k = 8 was selected as the number of topics reflecting interpretability and coherence [76]. The topic model was trained over 5000 Gibbs sampling iterations, with the initial 1000 iterations discarded as burn-in and every 100th sample retained to reduce autocorrelation. To induce sparsity in both the topic–document and word–topic distributions, the Dirichlet priors were set at α = 0.1 and δ = 0.01. Prior to modeling, we constructed a stopword list that extended standard English stopword collections with frequently occurring terms drawn from R&D-related texts, in order to eliminate non-informative vocabulary from the corpus.

## 4. Analysis Results

### 4.1. Descriptive Analysis

**Figure 2:**
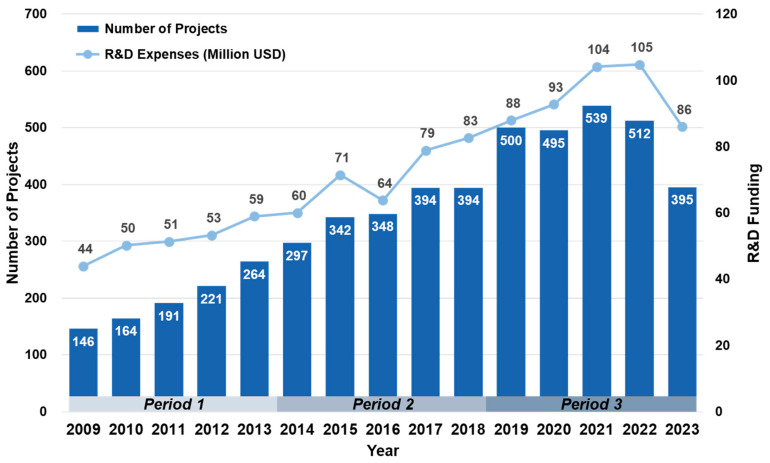
Statistics of biomimetic R&D projects.

From 2009 to 2023, South Korea funded 5202 biomimetic R&D projects, with a total investment of USD 1.09 billion (1 USD = 1148 KRW), as shown in Figure 2. The compound annual growth rate (CAGR) over this period was 4.9%. Funding increased steadily until 2022, but it declined sharply in 2023 due to reductions in the R&D budget, which affected project priorities and investment allocations. Of the total investment, USD 607.5 million was allocated to 3737 basic research projects at universities and research institutions, while USD 441.3 million supported 1342 projects focused on commercialization. On average, funding per project was higher for product development than for basic research. Biomimetic R&D projects were conducted over the long term, with 4033 lasting more than three years, which represented a substantial proportion of 77.5% of the total.

### 4.2. Interdisciplinary Research Network Analysis

**Figure 3:**
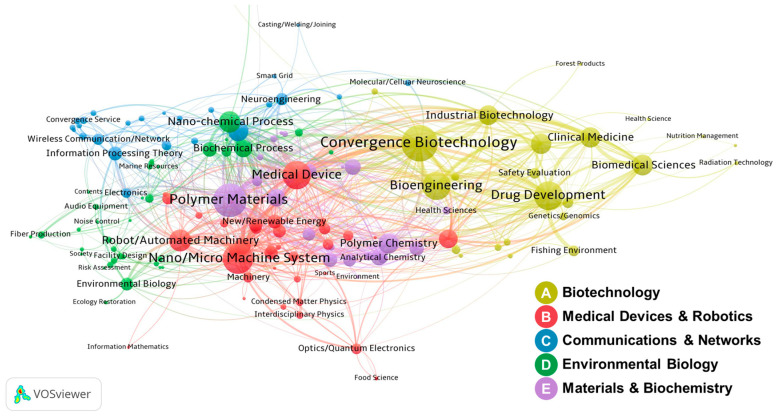
Interdisciplinary research co-occurrence network of research fields.

To identify interdisciplinary research fields in biomimetics, we analyzed a network structure comprising 151 research fields of NSTSC (intermediate classification) based on data from 2774 (53.3%) multidisciplinary convergence projects. Figure 3 presents the most frequently co-occurring research field pairs in convergence projects.

The research fields of “convergence biotechnology” and “biotechnology” co-occur most frequently and exhibit strong interactions with related interdisciplinary research fields. This network is associated with “convergence biotechnology”, “drug development”, “nano/micro-machine system”, “polymer materials”, “medical devices”, “nano-chemical processes”, “robot/automated machinery”, “semiconductor devices”, and “environmental ecology”.

The application of biomimetics across multiple disciplines, including materials science, engineering, medicine, and biology, is revealed by the cluster analysis. Specifically, cluster A (yellow) represents the intersection of biological sciences with various research fields and biological science, integrating biological principles with bioengineering, healthcare, and industrial biotechnology. Cluster B (red) primarily concerns the development of biomimetic medical devices and robotics, encompassing biosensors, drug delivery systems, biomimetic diagnostic tools, and therapeutic system designs. Cluster C (blue) examines the application of biomimetic methodologies in communication and networking technologies, with a particular emphasis on biomimetic algorithms for wireless networks, distributed sensor systems, and adaptive communication technologies, including neural networks and swarm intelligence. Cluster D (green) is concerned with environmental biology, namely nature-inspired solutions for environmental restoration and sustainable energy. Cluster E (purple) investigates the application of biomimetic materials and biochemistry, encompassing biomolecular systems, biocatalysts, polymers, and analytical chemistry. Collectively, these five clusters demonstrate how biomimetic convergence research advances both academic and commercial disciplines, including healthcare, robotics, materials, and environmental science.

The results of the centrality analysis for the biomimetic interdisciplinary research network are shown in Table 2**.** This analysis indicates that fields such as “convergence biotechnology”, “polymer materials”, “medical devices”, “nano/micro-machine system”, and “semiconductor devices”, exhibit consistently high centrality across all centrality metrics. These fields possess significant potential for interdisciplinary convergence, which can facilitate innovative product development and promote collaborative research. 

Degree centrality measures a node’s influence on its direct connections with other nodes. Nodes with high degree centrality exhibit the strongest links in research fields, which facilitate interconnection with other fields and provide insights into the diversity of interdisciplinary research. High-ranked degree centrality indicates extensive research on biocomposites, medical materials, nano-biomaterials, systems biology, and miniaturized sensors and devices. Closeness centrality evaluates a node’s direct and indirect influence by calculating its distance from all other connected nodes in the network. Research fields such as “drug development”, “semiconductor devices”, “nano-chemical processes”, and “robot/automated machinery” promote interdisciplinary convergence in biomimetics by enabling the rapid transmission and diffusion of information through the shortest connection paths.

Betweenness centrality identifies nodes that connect unlinked network nodes through the shortest paths, which play a crucial role in facilitating information flow and research collaboration. Fields with high betweenness centrality include “nano-chemical processes”, “semiconductor devices”, “drug development”, and “information theory”. The key fields, “polymer materials” and “information theory”, facilitate network interactions, strengthening connections across disciplines such as biotechnology and robotics. Eigenvector centrality quantifies a node’s importance based on its connections and influence. Fields such as “convergence biotechnology”, “medical devices”, “drug development”, and “biochemical process” foster collaboration and advance research in new drugs and medical devices within a network.

### 4.3. Keyword Analysis

Keywords, as essential textual elements, provide a concise overview of a paper’s important content and key points [100]. This section examines the most frequently used keywords and their co-occurrence patterns. It identifies changes in keyword trends by assuming that higher word frequency is associated with increased research interest in that field in each period. Furthermore, research trends are explored by quantifying keyword connectivity and relevance, examining keyword evolution, and identifying research structures [101].

#### 4.3.1. Keyword Frequency Analysis

This section analyzes the frequency of 5115 keywords extracted from 5202 R&D projects conducted between 2009 and 2023. To examine both commonly occurring and newly emerging keywords, the analysis period was divided into three five-year intervals: 2009–2013, 2014–2018, and 2019–2023 (Table 3).

The keyword frequency analysis indicates that the number of distinct words in biomimetic R&D projects increased over time, from 1755 to 3010. It can be inferred that new terms emerged as biomimetic research areas became more specialized and diverse. The most frequent word across all periods is “system”, followed by “material”, “nano”, “cell”, “3D”, “structure”, “drug”, “stem cell”, and “tissue”. These findings suggest that advanced nanotechnology and 3D printing play a critical role in the development of new composites, tissue scaffolds, nanocarriers, and medical devices that mimic biological structures and functions. The leading research fields in biomimetic R&D include systems biology, nanomaterials, cellular engineering, and regenerative medicine, with a specific focus on stem cell research and tissue regeneration. These trends reflect the government’s strategic investment in biotechnology (BT) and nanotechnology (NT) to advance biomimetic research.

The era-specific keywords indicate differentiated research trends in biomimetic R&D. In the first period (2009–2013), the keywords “robot”, “membrane”, and “actuator” appeared mainly in the first period. Research keywords related to biomimetic soft robots that mimic the behavior and sensing capabilities of living organisms are prominent. There has been active research into developing biomimetic robots, sensors, and actuators that mimic the locomotion, walking movements, and sensory capabilities of animals, insects, and fish. In addition, “energy”, “carbon”, and “environment” appeared as keywords representing carbon neutrality and environmental sustainability.

New keywords such as “disease”, “cancer”, “therapy”, “culture”, and “microenvironment” emerged in the second period (2014–2018). These keywords reflect a focus on addressing complex biological challenges in cancer treatment and cellular interactions. In other words, this period suggests that the growing importance or interest in biomimetic research applied to healthcare and cellular studies, which is grounded in the structures, systems, and characteristics of living organisms, has increased.

During the third period (2019–2023), keywords such as “treatment”, “organoid”, and “artificial intelligence (AI)” emerged concerning biomimetic research on biocompatible organs, scaffolds, organ-on-a-chip technologies, and bioinformatics. Furthermore, by examining the original content of R&D projects, it is evident that biomimetics has been applied to AI in various ways, including the development of algorithms, neural networks, computer vision, learning models, and robotics inspired by animal behaviors. In particular, AI technologies optimize the movement and adaptability of soft robots, while also enhancing the design and functionality of catalytic systems. Moreover, AI technologies optimize the movement and adaptability of soft robots and improve the design and functionality of catalytic systems. The presence of keywords such as “water”, “metal”, and “catalyst” indicates advancements in chemistry and materials science, particularly in chemical reactions, metal engineering, catalyst systems, and composites. Therefore, the keyword trends in biomimetic R&D have shifted towards more practical applications, particularly in healthcare, robotics, materials, and AI technologies.

#### 4.3.2. Keyword Network Analysis

**Figure 4:**
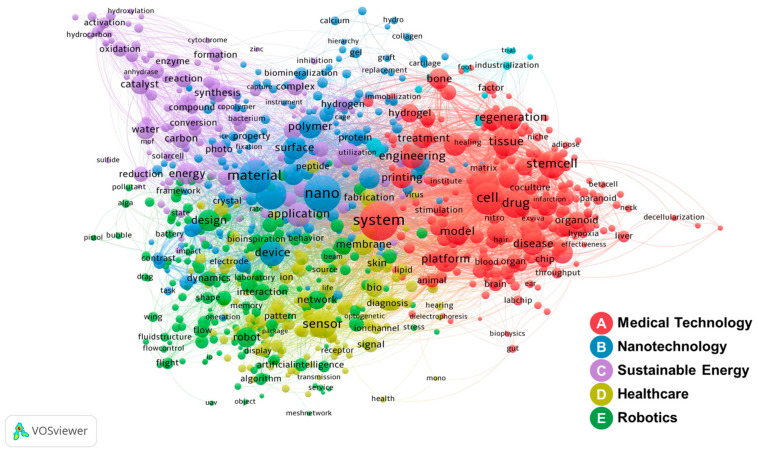
Keyword co-occurrence network (2009~2023).

The co-occurrence keyword network visualizes the interconnected relationships among key terms. As shown in Figure 4, frequently occurring terms are represented as nodes, and the connecting edges indicate the strength and frequency of their co-occurrence. The Louvain algorithm for modularity-based clustering identifies semantic groupings and structural patterns within the biomimetic R&D projects. The network comprises 875 keywords that appear more than ten times across the full dataset of biomimetic R&D projects.

The main keywords are comparable to the keyword frequency analysis results. This network establishes strong connections with drug delivery systems that mimic biological control and regulation mechanisms. In addition, it shows connections to sensors, robots, and actuators designed to imitate characteristics of animals, plants, and insects, as well as artificial organs, biocompatible materials, and nanomaterials suitable for human cells.

Cluster A (red) consists of R&D projects that apply biomimetics to advance healthcare systems and medical technologies. These projects explore biomimetic structures in tissue engineering to replicate the natural cellular environment and are related to research on drug delivery, disease modeling, and regenerative medicine. Cluster B (blue) represents the convergence of nanotechnology, materials, and device innovations, with an emphasis on developing lightweight, durable, and multifunctional nanostructures and surfaces. Within cluster C (purple), the high-priority biomimetic R&D projects focus on sustainability in energy, resource efficiency, and environmental remediation. This cluster investigates biomimetic solar panels, enzymatic reaction mechanisms, water purification, and management systems, as well as innovations in energy harvesting, chemical synthesis, and pollution control. Cluster D (yellow) emphasizes advances in adaptive and responsive technologies used in healthcare, robotics, and environmental monitoring. Key research focuses on biosensors for detecting biological changes, tactile sensors for human–robot interaction, biomimetic optical sensors, and flexible electronics for environmental real-time monitoring. Cluster E (green) deals with biomimetic robotics, control systems, and design methodologies that include human–robot interaction, soft robotics, and environmentally responsive robots for industrial automation and exploration. This keyword network shows a wide range of biomimetic applications from biomedical and robotics to nature-inspired solutions for renewable energy and clean environment systems.

Keyword centrality analysis reveals strong connections among different research terms. Table 4 presents centrality measures of the top 20 keywords, which exhibit high connectivity with other terms in the network. The high-ranking centrality values commonly include terms such as “system”, “nano”, “material”, “cell”, “structure”, and “control”. Moreover, keywords such as “application”, “surface”, “mechanism”, “model”, and “design” have relatively high centrality rankings compared to the frequency analysis results. These keywords pertain to the structural, functional, adaptive, and regulatory mechanisms observed in natural systems, including animals, plants, insects, and ecosystems.

In the network, keywords with high degree centrality also present high closeness centrality. These keywords are involved in the application of biomimetic principles to robots, tactile sensors, nano-devices, and control technologies. The network includes biopolymer scaffolds for human tissue and organ regeneration in healthcare, as well as drug delivery systems and tissue engineering for disease treatment. Furthermore, keywords with high betweenness centrality significantly influence surface treatment technologies, biomaterials, robotic designs, and sensor technologies, as they mediate the flow of information within the network. Finally, eigenvector centrality reflects the prominence of dominant terms, including biosensors, nano-polymers, and device designs. Therefore, keywords have high interconnectivity with various terms that contribute to the expansion and integration of research areas. However, the network structure does not clearly show connections between keywords related to specific organisms.

### 4.4. Topic Modeling Analysis

This section applies LDA topic modeling to cluster biomimetic research topics and analyzes changes over time. Hierarchical clustering measures the similarity of topics and reveals their interrelationships and distinguishing features.

#### 4.4.1. Analysis of Research Topics

The analysis of LDA topic modeling identified eight research topics in biomimetic R&D projects. Table 5 shows a dendrogram structure of research topics categorized into three clusters. Biomimetic R&D is divided into three clusters: intelligent robotics (Cluster 1), biomedical engineering (Cluster 2), and materials science (Cluster 3).

Cluster 1 includes “biomimetic computing (Topic 1)” and “robotics and fluid dynamic systems (Topic 2)” that mimic biological processes for optimized motion and decision-making. Topic 1 pertains to biologically inspired computing, which uses biological systems and processes as models for computational methods and systems design. It emphasizes biomimetic research that draws inspiration from biological systems to develop sensors, networks, and AI algorithms for efficient detection, signal processing, pattern recognition, and control systems. Moreover, biomimetic approaches are being studied to improve system efficiency through optimization or heuristic algorithms inspired by the collective behavior of biological groups such as ants, bees, birds, and fish. Topic 2 involves robotics and dynamic systems inspired by biological processes in physical design, actuator systems, efficient motion control, fluid dynamics, and energy optimization. These technologies enhance the functionality and performance of robotics that emulate natural traits of animals, such as the aerodynamic efficiency of bird wings, the agility of fish swimming, and the precise, energy-efficient movements of insects. These biomimetic technologies enhance functionality and adaptability and are applied in fields such as mechatronics, aerospace, drones, autonomous vehicles, and underwater robotics.

Cluster 2, which accounts for the largest share of research funding, includes “tissue engineering and regenerative medicine (Topic 3)”, “drug delivery systems (Topic 4)”, and “drug screening platforms (Topic 5)”. This cluster indicates an interdisciplinary approach, combining biology, engineering, and medicine principles to develop technologies and devices that solve healthcare problems. Topic 3 involves tissue engineering for organ regeneration, wound healing, and stem-cell therapies, which are applied to the treatment of damaged tissues and the advancement of regenerative medicine. Artificial tissues replicate the structural and functional characteristics of tissues by integrating various approaches, such as 3D printing, scaffolds, hydrogels, biomaterials, and phototherapy. Topic 4 explores biomimetic drug delivery systems that employ nanoparticles and biomaterials to precisely target tumors or diseased tissues. These systems are designed to navigate the tumor microenvironment, replicate cellular interactions, and achieve receptor-mediated targeting, which improves treatment efficacy and minimizes side effects in cancer and genetic diseases. Topic 5 focuses on the development of biomimetic drug screening platforms to evaluate drug efficacy, safety, and toxicity using human disease models. This research emphasizes advanced systems, including organoids, membrane cultures, and biochips, which replicate disease conditions to improve drug testing accuracy. Thus, cluster 2 can contribute to drug discovery, disease modeling, and advancements in precision medicine by simulating various physiological processes in the human body.

Cluster 3 integrates three core topics into “catalytic systems (Topic 6)”, “material synthesis and design (Topic 7)”, and “surface engineering (Topic 8)”. These topics apply to sustainable energy and materials innovative solutions. Topic 6 explores biomimetic catalysis, efficient energy conversion, carbon dioxide (CO_2_) reduction, purification systems, and oxidation-reduction reactions. This topic emphasizes the implementation of efficient chemical reactions by mimicking natural catalytic processes. Biomimetic catalysis encompasses the emulation of biological catalytic processes found in natural systems, such as enzymatic reactions, photosynthetic mechanisms, redox processes, and decomposition pathways. It is applied in eco-friendly technologies, including carbon capture and utilization systems (CCUS), hydrogen production, organic compound synthesis, and environmental purification. Topic 7 focuses on the design and synthesis of nanostructured and hybrid materials. This research explores how polymers, metals, and self-assembly mechanisms can be strategically combined for applications in energy storage, catalysis, sensors, and optoelectronics. Topic 8 involves the development of coatings, hydrogels, and nanostructures that replicate the functional characteristics of natural fibers and surfaces. The principles of biomineralization and biological adhesion are applied to create multifunctional materials that replicate the natural durability, adaptability, and functionality. These materials are inspired by lotus leaves for superhydrophobic coatings, shark skin for anti-fouling surfaces, geckos and mussels for adhesion, and desert plants for water-harvesting capabilities. This cluster draws on multidisciplinary knowledge from chemistry, biology, and engineering to develop materials with properties inspired by natural organisms.

#### 4.4.2. Trends Analysis of Research Topics

**Figure 5:**
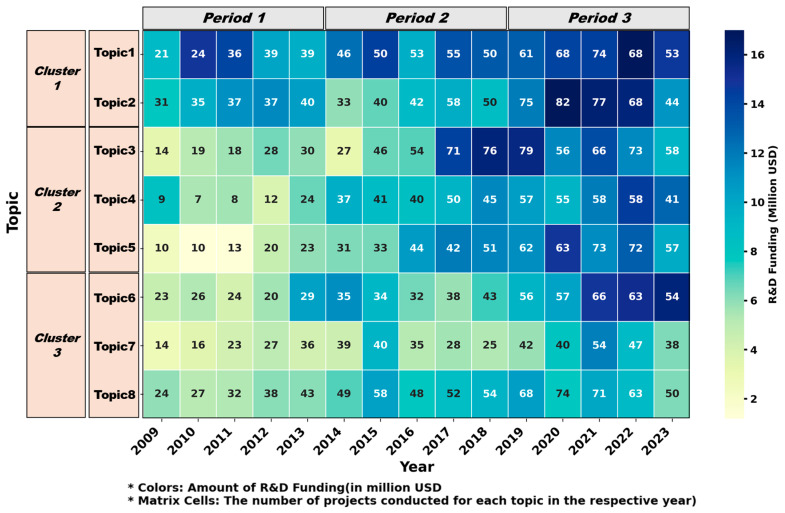
Distribution of research funding and projects by year and topic (2009~2023).

The trends in research topics and the distribution of projects in biomimetic R&D across three distinct periods are shown in Figure 5. This matrix indicates the funding levels, number of projects, and temporal shifts associated with each topic.

Some topics (Topic 1, Topic 4, and Topic 6) appear to attract more funding relative to their project count, which suggests that they are higher investment priorities or require more capital-intensive resources. Although topic 8 has the highest number of projects, its funding is lower than that of other topics. This indicates that topic 8 involves lower-cost projects or that the projects have smaller funding allocations. Topic 7 is the least-funded and least-researched area in biomimetics, which may reflect its current status as a lower-priority area or indicate the necessity for cost-effective advancements in the future.

The first period (2009–2013) was focused on establishing the foundational research for biomimetic robotic systems and dynamic engineering solutions through the study of biological motion and structures. Research investment was directed toward biomimetic computing models, biomimetic algorithms, and fluid dynamic systems, which became integral to the evolution of autonomous robotics. Furthermore, biomimetic research expanded into fields such as biomedical engineering and materials science. Biomimetic approaches were studied in tissue scaffolding, regenerative medicine, and targeted drug delivery in healthcare and gained attention due to their potential therapeutic applications. As “blue technology” emerged as a significant game changer for sustainable development, research shifted toward biomimetic multifunctional materials, self-healing materials, and eco-friendly catalysts.

The second period (2014–2018) represented a shift from foundational exploration to applied research, accompanied by a substantial increase in projects and research funding in biomedical engineering. This period was characterized by the development of biomimetic medical devices, sustainable biomaterials, personalized drug delivery systems, and nanotechnology-driven regenerative medicine within the precision medicine framework. Materials science experienced sustained growth, driven by the government’s strategic support for nanotechnology initiatives to develop sustainable materials. Researchers focused on implementing biomimetic hybrid materials in various industrial applications, including high-efficiency solar cells, air filtration systems, smart fabrics, and construction.

In recent years (2019–2023), biomimetic research has invested significantly in practical applications across a variety of disciplines, with a particular focus on interdisciplinary convergence and integration of biomimetic principles and AI. There has been a notable increase in funding and advancements in biomimetic robotics [4,45,46,102], optimization algorithms [103,104,105], and AI systems [106]. In particular, AI models have been used to analyze the variables affecting catalyst performance and to develop innovative catalysts that enhance reaction efficiency, energy conversion, and CO_2_ reduction. The convergence of biomimetics with computing methods has facilitated the development of diverse AI-driven applications, including autonomous systems, medical diagnostics, pattern recognition, and eco-friendly chemical processes.

### 4.5. Technology-Product Implementation

**Figure 6:**
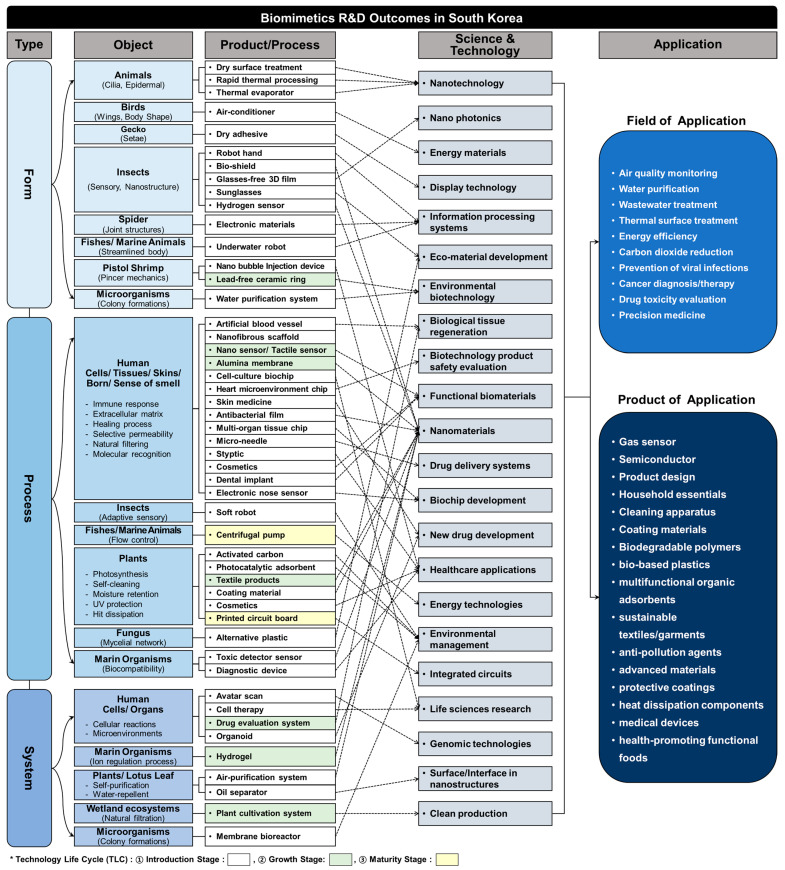
Topology of commercialization in biomimetic technologies.

To understand the pathways through which research outcomes are translated into commercial products, the topology of biomimetic R&D projects is analyzed. It sheds light on the market potential of biomimetic technologies. The commercialization of biomimetic R&D is examined by categorizing projects based on biomimetic types, objects, scientific domains, stages within the technology lifecycle (TLC), products, and future potential (Figure 6).

Implementing biomimetic principles in practical solutions frequently encounters challenges, such as high prototype development costs, limitations in current manufacturing techniques, and complexities in crafting effective business strategies for market entry [107]. Despite these barriers, approximately 6% of biomimetic R&D projects (326 cases) have successfully transitioned to commercialization. Biomimetic products are designed to replicate natural forms, processes, and systems [1].

The results of this analysis revealed ten key biomimetic commercial products: nanosensors, robots, adhesives, electronic skin, hydrogels, cosmetics, air purifiers, water treatment systems, coating materials, and textiles. These key products were inspired by diverse biological mechanisms, including the sensory systems of insects [108], the movements of animal cilia [109], the adaptability of human tissues [3], plant-based self-purification processes [110], and the fluid dynamics observed in marine organisms [111]. These biomimetics-inspired products have contributed to drug screening [112], carbon reduction, water and air quality improvement, pollution management [113], and advanced composite materials [114,115]. However, most biomimetic products still remain in the introduction stage of the technology life cycle (TLC), while only seven products are advancing to the growth stage and, finally, two reach the maturity stage. To ensure that innovation aligns with specific industrial needs and achieves widespread applicability, strategies are required to address the challenges of commercialization and market integration.

## 5. Discussion and Conclusions

This paper presents an in-depth analysis of 5202 national R&D projects on biomimetics in South Korea from 2009 to 2023. Network analysis and topic modeling were performed on biomimetics to examine changes in research trends and characteristics across different periods and interdisciplinary fields. These analyses were conducted by extracting keywords and research field information from the content of each project. The interdisciplinary research and keyword analysis sections highlight the important research areas of activity and identify the most prominent keywords in the respective period. This work quantifies the research activity on biomimetics for convergence collaborations across different disciplines, analyzes how it has changed over time, and describes topic areas as a leading edge ongoing toward biomimetics applications.

The results of this study are summarized in the following four points: (1) Over the past 15 years, biomimetic research has shifted from biomimetic computing for fundamental mechanisms to tissue engineering and regenerative medicine for therapeutics, and has recently detected a rapid change in trend toward AI-driven applications in various biomimetic fields. (2) Like other fields, biomimetic research shows an innovation gap between technology and business. (3) An increasing tendency toward multidisciplinary and convergence-oriented biomimetic research is noted. (4) Public research funding and projects have significantly increased for AI-related algorithms, robots, and systems.

Our results, through the analyses of keyword frequency and topic modeling, reveal biomimetic R&D trends in each period. In the first period (2009–2013), biomimetic research can be summarized by a predominant focus on biomimetic computing (Topic 1), along with membranes, actuators, and fluid dynamics. This period was characterized by biomimetic mechanical systems and fundamental engineering research. During the second period (2014–2018), biomimetic R&D has expanded into healthcare domains, accompanying a substantial increase in tissue engineering and regenerative medicine (Topic 3) in aspects of financial support and project frequency. The third period (2019–2023) has been characterized by diverse AI-driven applications integrated with biomimetics. In addition, in this period, research fields have exponentially expanded through biomimetic computing (Topic 1), regenerative medicine (Topic 3), and biomimetic catalysis (Topic 6). This implicates a progressive shift toward applied biomimetics, demonstrating profoundly enhanced industrial applications in intelligent robotics, materials science, biomedical engineering, and biomimetic AI development.

Of note, through the investigated periods, we noticed a growing trend in biomimetic developments responding to societal and environmental problems around the world. The ability of biomimetics to effectively address social needs and challenges is critically important in its uptake and further development of applications. In fact, the nature of biomimetics by definition is derived from nature resources and/or nature-mimicking inspiration. Also, importantly, this characteristic nature of biomimetics itself can potentially contribute to developing sustainable and bio-compatible solutions for societal and environmental challenges to overcome. Our results show that a considerable number of developing technologies concerning these sustainable and biocompatible solutions are emerging toward the growth and maturity stages, moving beyond the introduction stage (Figure 6). For example, as technologies reach the growth and maturity stages, lead-free ceramic rings, centrifugal pumps, and plant cultivation systems (Figure 6) are now being substantially developed for environmental biotechnology, energy efficiency, water purification, and carbon dioxide reduction toward industrial applications. These growing trends in biomimetics might indeed be on a promising pathway for advancing the development of sustainable technologies in the near future.

Our analysis also unveiled the discrepancy between basic research in biomimetics and its concrete application, e.g., “innovation gap” known in the literature [116]. Based on our results, while government funding for biomimetic R&D remains primarily focused on basic research conducted at universities, there are far fewer applied research projects aimed at full-scale product development and commercialization. As shown in the topology analysis results for verifying the path leading to commercial products, only about 6% of biomimetic R&D projects have been commercialized. On the contrary, the fact that the average funding per applied research project is nearly twice that of basic research projects implies that basic biomimetic research still primarily focuses on conceptual exploration with limited industrial linkage, while public funding is selectively concentrated on only a small number of applied research projects. This indicates that biomimetic fields also confront the innovation gap that often occurs in the innovation process from basic research to applied research and commercialization. In order to reduce this gap, it is necessary to reconstruct the national innovation strategy by weighted selection and support for emerging key research areas, and by rebalancing the funding support according to innovation stages. For this, a classification system for optimized national biomimetic R&D support can be suggested through re-evaluating each biomimetic field according to innovation stages. In keeping with these national strategies, at the industrial level, an ecosystem to strategically foster new industries potentially provoked by biomimetic breakthroughs needs to be established to create a market with new business models at the corporate or individual level. As an encouraging aspect, our results show a progressive shift toward applied biomimetics during recent years, which demonstrates increasing industrial applications in intelligent robotics, materials science, biomedical engineering, and bioinspired algorithm development. Stimulations via well-organized national funding are essential to support technological breakthroughs in biomimetic R&D, which enable researchers to traverse the valley of death and advance toward successful innovation and commercialization.

According to our co-occurrence network analysis, biomimetic convergence research is shown to be mainly concentrated in biotechnology, materials, and robotics. These convergence areas play a critical role in promoting research collaboration across adjacent or disparate scientific disciplines. A recent decade of achievements in biomimetic technologies clearly shows that their advancement requires a high level of interdisciplinary capability that bridges engineering design with technological implementation. This multidisciplinary approach allows for the enhancement of the practical effectiveness of the technology, as well as its potential for commercialization and the development of a robust industrial ecosystem. Considering this increasing tendency toward interdisciplinary biomimetic convergence, government strategies for supporting key biomimetic research fields should be carefully established in order to achieve well-balanced research funding investments across key research fields, which could promote interdisciplinary research projects reinforcing a public–private, industry–academia research cooperation network. This interdisciplinary biomimetic support by the government can greatly contribute to improving product functions and performance through enhanced biomimetic technologies by biomimetic convergence research, as well as aiding the development of new business models.

Our research topic trend analysis shows that research funding and projects have significantly increased for AI-related algorithms, robots, and systems. For example, biomimetic AI applications via biomimetic image and speech processing enable more accurate replication of specific sense organ functions [117,118]. This convergence is anticipated to significantly enhance interdisciplinary research efforts and catalyze innovative scientific paradigms at this emerging intersection. AI-based digital twin system simulations, which are actively being conducted in the manufacturing and new product development fields, will be applied to improve the efficiency of shortening the research period and cost in multidisciplinary and convergence research projects. In other aspects, regarding the fact that a vast amount of ecological information is currently dispersed, an AI-based ecological approach can be effectively and easily utilized for solving the existing technological problems in biomimetic R&D. The integration of machine learning, empirical modeling, and computational simulations can deepen our understanding of biological systems and evoke simultaneous acceleration of development timelines in nature-inspired technologies [119,120,121]. AI-integrated biomimetic technology may play a critical role in developing advanced manufacturing techniques that more accurately replicate the intrinsic properties of organisms and ecosystems in the near future, indicating that AI-driven research may accelerate current convergent research trends in biomimetics. A national initiative for AI-driven biomimetic research can suggest strategies for cost-effective biomimetic R&D management.

Although this study derived meaningful biomimetics trends and implications for future directions, some limitations are worth noting. Since this study only analyzed data from South Korea’s R&D projects, it does not capture global research trends and variations in biomimetics across different countries. Hence, future studies should expand to include international datasets, particularly cross-country analyses of R&D projects or patents, to provide a comparative perspective on global trends and their progression. Additionally, it is necessary to analyze R&D projects supported by regional and private sectors in future studies, which will further help to gain a more in-depth understanding of industry trends in specific research topics and innovation patterns in biomimetics.

In conclusion, the results of the present study suggest the following trends in biomimetic research and national biomimetic R&D policies: (1) Key driving factors for the evolution of biomimetic research have changed over time. Although biomimetic evolution still remains primarily focused on basic research, the trends have recently progressively shifted toward applied biomimetics. (2) Biomimetic convergence and interdisciplinary research are rapidly growing. (3) Sustainable and bio-compatible biomimetic fields are now in part reaching growth and maturity stages. (4) A classification system for optimized national biomimetic R&D support can be suggestive for re-evaluating each biomimetic field according to its innovation stages. Overall, the present analytic results may provide policymakers and enterprises with insight into the evolution and outlook of biomimetic R&D.

## Figures and Tables

**Table 1:** Comparison of topic models.

Comparative Models	LDA	NMF	TF-IDF	BERTopic
Dimensionality reduction	V	V	V	V
Semantic Annotation	V	V		V
Mixture Model	V			
Generation Ability	V			V
Scalability	V	V	V	
Computational Efficiency		V	V	
Interpretability	V	V	V	
Stability for Large Database	V	V	V	V

**Table 2:** Top 10 centrality indices of an interdisciplinary research network.

Rank	Degree	Closeness	Betweenness	Eigenvector
1	Convergence Biotechnology (0.387)	Convergence Biotechnology (0.588)	Convergence Biotechnology (0.588)	Convergence Biotechnology (1.000)
2	Polymeric Materials (0.367)	Polymeric Materials (0.588)	Polymeric Materials (0.100)	Polymeric Materials (0.992)
3	Medical Devices (0.333)	Medical Devices (0.560)	Medical Devices (0.092)	Medical Devices (0.868)
4	Nano/Micro Machine System (0.307)	Nano/Micro Machine System (0.560)	Nano/Micro Machine System (0.071)	Nano/Micro Machine System (0.842)
5	Drug Development (0.267)	Drug Development (0.540)	Semiconductor Devices (0.071)	Nano-chemical Processes (0.767)
6	Nano-chemical Processes (0.260)	Semiconductor Devices (0.540)	Drug Development (0.060)	Drug Development (0.753)
7	Semiconductor Devices (0.260)	Nano-chemical Processes (0.538)	Information Theory (0.056)	Semiconductor Devices (0.686)
8	Robot/Automated Machinery (0.207)	Robot/Automated Machinery (0.519)	Environmental biology (0.050)	Biochemical Process (0.643)
9	Materials Chemistry (0.207)	Molecular Cell Biology (0.512)	Manufacturing Platform (0.047)	Materials Chemistry (0.627)
10	Biochemical Process (0.207)	New/Renewable Energy (0.508)	Bioengineering (0.045)	Metallic Materials (0.620)

**Table 3:** Keyword frequency analysis in R&D projects.

All-Time (Top 20)			Common			New Emergence (Top 50)	
Rank	Word	Freq.	1P	2P	3P	Period	Word (Freq.)
1	System	1155	○	○	○	1P (2009~2013)	Robot (81) Energy (68) Membrane (56) Actuator (53) Environment (47) Carbon (45)
2	Material	722	○	○	○		
3	Nano	687	○	○	○		
4	Cell	639	○	○	○		
5	3D	558	-	○	○		
6	Structure	512	○	○	○		
7	Drug	487	○	○	○		
8	Stem cell	466	○	○	○	2P (2014~2018)	Network (90) Disease (83) Therapy (83) Screening (80) Cancer (76) Culture (74) Microenvironment (74)
9	Tissue	458	○	○	○		
10	Device	423	○	○	○		
11	Sensor	404	○	○	○		
12	Control	375	○	○	○		
13	Application	361	○	○	○		
14	Platform	341	-	○	○		
15	Polymer	335	○	○	○	3P (2019~2023)	Treatment (133) Organoid (133) Water (114) Metal (111) Catalyst (93) Artificial Intelligence (76)
16	Model	330	-	○	○		
17	Regeneration	329	○	○	○		
18	Engineering	320	○	○	-		
19	Surface	313	○	-	○		
20	Design	291	○	-	-		

**Table 4:** Top 20 words of keyword network centrality (2009~2023).

Rank	Degree	Closeness	Betweenness	Eigenvector
1	System (0.346)	System (0.604)	System (0.127)	System (1.000)
2	Nano (0.235)	Nano (0.566)	Material (0.057)	Nano (0.813)
3	Material (0.222)	Material (0.562)	Nano (0.056)	Material (0.784)
4	Cell (0.210)	Cell (0.557)	Cell (0.044)	Cell (0.747)
5	Structure (0.184)	Structure (0.549)	Structure (0.038)	Structure (0.694)
6	3D (0.171)	3D (0.546)	Mechanism (0.034)	3D (0.676)
7	Control (0.162)	Control (0.543)	Control (0.030)	Control (0.658)
8	Application (0.154)	Application (0.540)	Application (0.028)	Drug (0.635)
9	Drug (0.153)	Mechanism (0.540)	Sensor (0.027)	Application (0.635)
10	Mechanism (0.151)	Device (0.539)	Device (0.026)	Device (0.625)
11	Sensor (0.149)	Drug (0.539)	3D (0.026)	Model (0.605)
12	Device (0.148)	Sensor (0.538)	Drug (0.021)	Platform (0.595)
13	Tissue (0.140)	Model (0.535)	Surface (0.020)	Mechanism (0.586)
14	Model (0.139)	Surface (0.534)	Model (0.020)	Sensor (0.584)
15	Surface (0.134)	Tissue (0.533)	Design (0.020)	Polymer (0.574)
16	Platform (0.130)	Platform (0.533)	Robot (0.019)	Tissue (0.570)
17	Stem-cell (0.127)	Design (0.531)	Tissue (0.019)	Engineering (0.567)
18	Design (0.125)	Engineering (0.531)	Network (0.018)	Surface (0.537)
19	Engineering (0.125)	Stem-cell (0.530)	Platform (0.017)	Stem-cell (0.529)
20	Robot (0.114)	Hybrid (0.527)	Polymer (0.016)	Hybrid (0.529)

**Table 5:** Biomimetics Topic Clusters and Keywords.

Cluster/Topic		Topic Name/Keywords (Top 10)	Projects (%)	Expenses (M USD, %)
Cluster 1 (Intelligent Robotics)	Topic 1	[Biomimetic computing] sensor, system, network, signal, processing, control, detection, pattern, recognition, electronics, robot, algorithm, transistor, membrane, artificial intelligence (AI)	737 (14.2%)	194.0 (17.8%)
	Topic 2	[Robotics and fluid dynamics systems] robot, design, actuator, sensor, fluid, flow, dynamics, flight, shape, motion, control, energy, optimization, artificial intelligence (AI), vehicle	749 (14.4%)	161.9 (14.9%)
Cluster 2 (Biomedical Engineering)	Topic 3	[Tissue engineering and regenerative medicine] tissue, regeneration, stem-cell, 3d, printing, scaffold, engineering, born, treatment, biomaterial, hydrogel, device, differentiation, phototherapy, mechanism	715 (13.7%)	135.1 (12.4%)
	Topic 4	[Drug delivery systems] drug, system, delivery, cancer, disease, cell, therapy, tumor, tissue, gene, treatment, medicine, microenvironment, target, nanoparticle	542 (10.4%)	136.2 (12.5%)
	Topic 5	[Drug screening platforms] drug, cell, screening, platform, disease, model, efficacy, evaluation, organoid, membrane, culture, safety, biochip, toxicity, test	604 (11.6%)	122.3 (11.2%)
Cluster 3 (Materials Science)	Topic 6	[Catalytic systems] system, water, catalyst, energy, CO_2_, oxidation, membrane, reaction, reduction, conversion, metal, production, enzyme, purification, carbon	600 (11.5%)	135.4 (12.4%)
	Topic 7	[Materials synthesis and design] material, synthesis, application, design, energy, polymer, nanostructure, metal, hybrid, reaction, chemistry, control, catalyst, assembly, complex	504 (9.7%)	90.5 (8.3%)
	Topic 8	[Surface engineering] material, polymer, surface, coating, system, hybrid, structure, hydrogel, fabrication, biomineralization, adhesion, application, metal, fiber, nanoparticle	751 (14.4%)	114.7 (10.5%)

## Data Availability

The presented in this study are available from the corresponding author upon reasonable request.
